# Retraction: Oral administration of brain protein combined with probiotics induces immune tolerance through the tryptophan pathway

**DOI:** 10.3389/fnmol.2023.1279716

**Published:** 2023-08-29

**Authors:** Frontiers Editorial Office

The journal retracts the 28 May 2021 article cited above.

Following publication, concerns were raised regarding the integrity of the images in the published figures. Upon further assessment, Image duplication was identified in Figures 2F and 3I and Figures 2B and 3C.

The authors were responsive to our queries during the investigation, which was conducted in accordance with Frontiers' policies. However, they were unable to provide a satisfactory explanation during the investigation that allowed us to verify the data and conclusions presented. As a result, the data and conclusions of the article have been deemed unreliable and the article has been retracted.

This retraction was approved by the Chief Editor of Frontiers in Molecular Neuroscience and the Chief Executive Editor of Frontiers. The authors did not agree to this retraction.

